# Pulmonary Alveolar Proteinosis in an Infant with ADA-Deficient SCID †

**DOI:** 10.3390/pediatric18050141

**Published:** 2026-10-21

**Authors:** Léa El Haddad, Mark Thomas Vander Lugt, Thomas G. Saba

**Affiliations:** Department of Pediatrics, University of Michigan, Ann Arbor, MI 48109, USA

**Keywords:** Adenosine deaminase-deficient severe combined immunodeficiency (ADA-SCID), pulmonary alveolar proteinosis (PAP), hematopoietic stem cell transplantation (HSCT)

## Abstract

Adenosine deaminase-deficient severe combined immunodeficiency (ADA-SCID) is a rare disorder characterized by profound lymphocytopenia, recurrent infections, and non-infectious complications such as pulmonary alveolar proteinosis (PAP). PAP in ADA-SCID results from impaired surfactant clearance due to dysfunctional alveolar macrophages and may cause hypoxemia, chronic pulmonary complications and death if treatment is delayed. This case describes a 3-week-old female diagnosed with ADA-SCID, who developed acute hypoxic respiratory failure secondary to PAP confirmed by positive Periodic Acid–Schiff staining of bronchoalveolar lavage and diffuse ground glass opacities on chest CT. The child was treated with enzyme replacement therapy (ERT) and hematopoietic stem cell transplantation (HSCT) and demonstrated normalization of physical exam, oxygen saturation and chest imaging. This case illustrates the importance of early recognition and definitive therapy for ADA-SCID-associated PAP including ERT and HSCT, rather than traditional and high-risk interventions for pediatric PAP including whole lung lavage. Timely intervention can lead to favorable pulmonary outcomes.

## 1. Introduction

Adenosine deaminase-deficient severe combined immunodeficiency (ADA-SCID) is a rare, autosomal recessive disorder of purine metabolism that results in profound impairment of both humoral and cellular immunity [1]. The incidence in the US is approximately 1 case per 500,000 births [2]. The accumulation of toxic purine metabolites leads to marked lymphocytopenia and severe immunodeficiency, typically presenting in early infancy with recurrent infections, failure to thrive, and absence of lymphoid tissue. In addition to immune dysfunction, ADA-SCID is associated with a spectrum of non-immune manifestations, including skeletal abnormalities, neurodevelopmental delay, and pulmonary complications [1]. In particular, approximately 50% of patients with ADA-SCID develop pulmonary alveolar proteinosis (PAP). Delayed treatment of PAP has been associated with chronic pulmonary complications and death [3]. This report describes a case of ADA-SCID complicated by PAP successfully treated with enzyme replacement therapy and allogenic hematopoietic stem cell transplantation, rather than traditional and risky pediatric PAP therapies including whole lung lavage.

## 2. Case Presentation

### 2.1. Patient Background

A 3-week-old female, born at 39 weeks via spontaneous vaginal delivery was diagnosed with ADA-deficient SCID based on newborn screening (undetectable TREC, elevated d-ADO: 14.912 µmol/L, normal <0.2 µmol/L). Confirmatory genetic testing showed compound heterozygous mutations in the *ADA* gene (c.632G>A; c.956_960del). Lymphocyte subsets showed the absence of T, B, and NK cells. ADA enzyme activity was undetectable (0.2 nmol/h/mg; normal >60), and toxic metabolites (dAXP 1.444 µmol/mL) were markedly elevated. Maternal engraftment was not detected. Following diagnosis, the patient was followed closely as an outpatient with plans to start enzyme replacement therapy with elapegademase as a bridge to allogeneic hematopoietic stem cell transplant (HSCT).

### 2.2. Initial Presentation and Hospital Course

**Figure 1:**
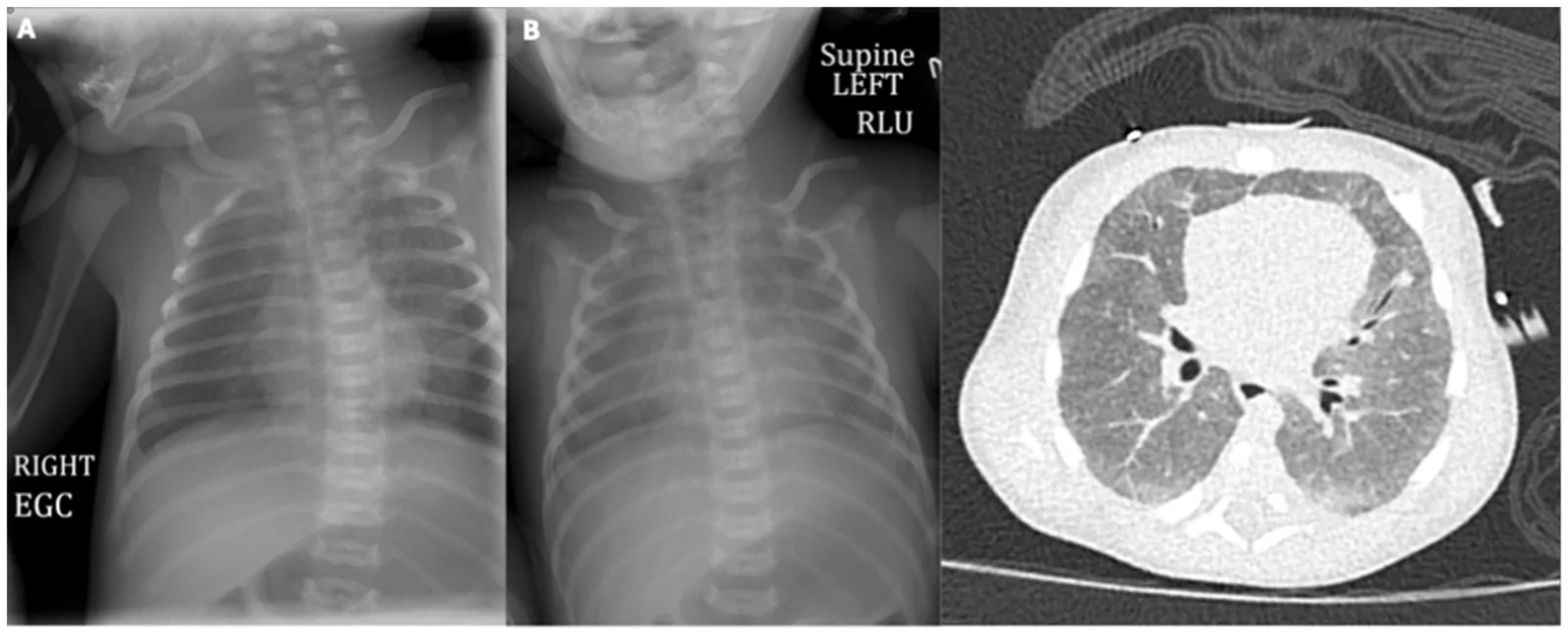
(**A**) Chest X-ray upon admission showing normal lung volumes without significant opacification. (**B**) Chest X-ray and CT scan 1 week after admission show diffuse ground glass opacities throughout the lungs with mild mosaic attenuation and without evidence of septal thickening.

At 3 weeks of age, the patient presented to the emergency department with acute hypoxic respiratory failure. On exam, she was tachypneic with respiratory rate ranging between 48 and 76 breaths per minute, had subcostal, intercostal and supraclavicular retractions, and head bobbing. No stridor or wheezing was appreciated on exam, and lungs were clear to auscultation. Initial chest X-ray showed low lung volumes but no concerns for pleural effusion or consolidation (Figure 1A).

Venous blood gas showed no evidence of metabolic or respiratory gas exchange abnormalities. She required support with a heated high flow nasal cannula for dyspnea. She was started on enzyme replacement therapy (ERT) with elapegademase (0.2 mg/kg IM twice weekly) shortly after admission to help with immune reconstitution given concerns for infection.

The family reported three episodes of self-resolving apnea lasting 10–15 s over 5 days prior to hospital admission. Additional symptoms included poor feeding and non-bilious, non-bloody projectile emesis. The child was empirically started on antibiotics. Sepsis and other infections were ruled out. Viral polymerase chain reaction was negative.

A video fluoroscopic swallow study showed laryngeal penetration. The patient was then started on a thickened formula to reduce the risk of aspiration. The patient was also started on empiric airway clearance to help mobilize mucus and reduce the risk of atelectasis.

**Figure 2:**
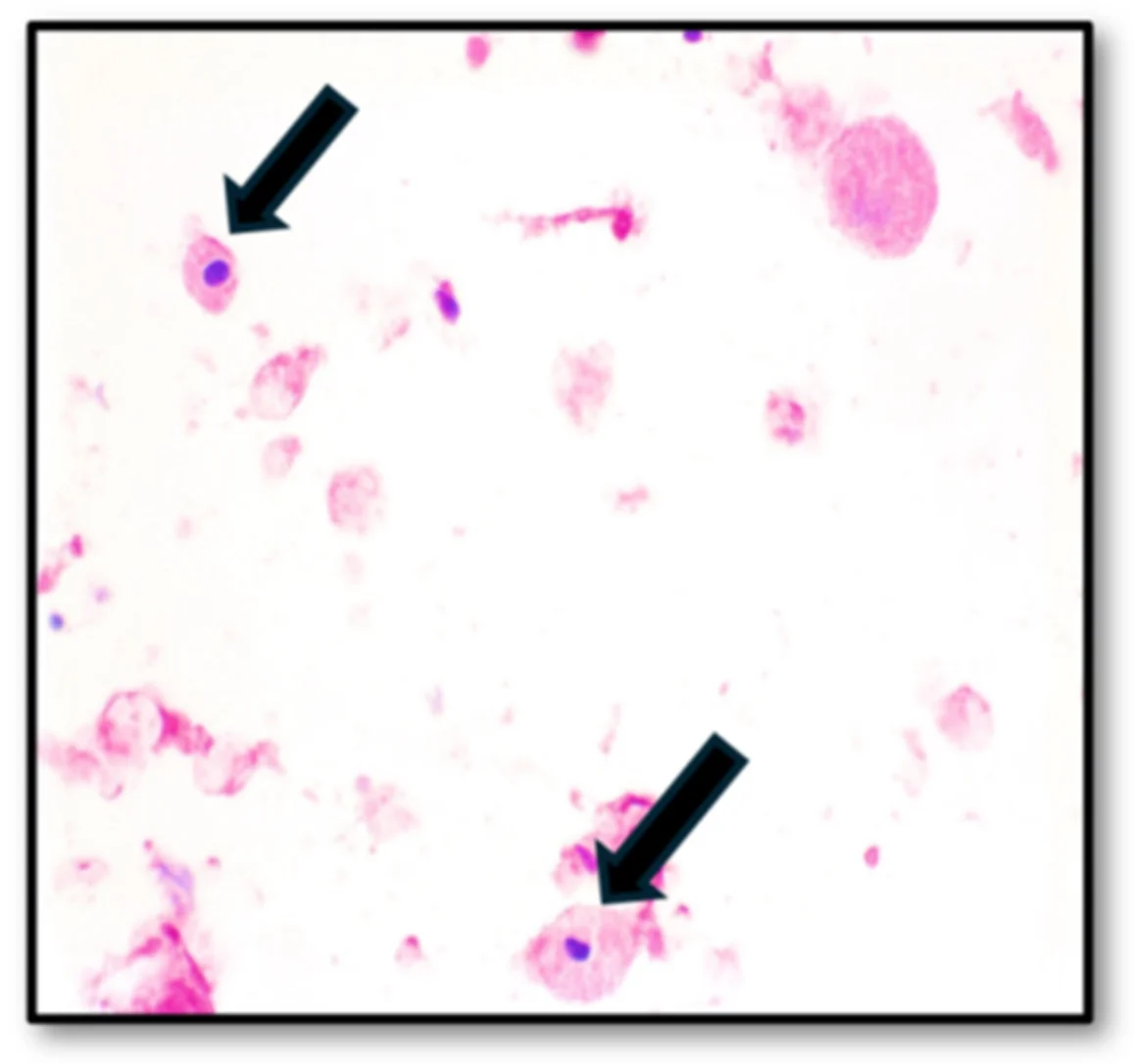
Cytology on bronchoalveolar lavage fluid showing PAS-positive, diastase-resistant granular proteinaceous deposits within alveolar macrophages (arrow), suggestive of pulmonary alveolar proteinosis.

Despite these supportive measures, the patient’s respiratory failure worsened, and she required endotracheal intubation. A chest X-ray one week after presentation showed new, diffuse opacification; high-resolution computed tomography (HRCT) scan showed diffuse ground-glass opacities with mild mosaic attenuation, concerning for PAP or atypical infection (Figure 1B). Bronchoscopy with bronchoalveolar lavage (BAL) revealed milky-appearing fluid, with PAS-positive granular material suggestive of PAP (Figure 2).

Infectious studies including *Pneumocystic jiroveci* polymerase chain reaction, galactomannan, respiratory virus panel, and bacterial cultures were negative. On the day of bronchoscopic evaluation, the ERT dose was increased to 0.4 mg/kg. Her respiratory status subsequently improved markedly over the following days; however, given the close temporal relationship between the dose escalation, bronchoscopic evaluation, and clinical improvement, the specific contribution of the dose increase to her improvement cannot be determined. However, since only 30 mL were used for BAL, it is unlikely to have offered significant therapeutic benefit.

Within two days, the patient was successfully extubated directly to the nasal cannula. She continued to improve clinically, was weaned off supplemental oxygen, and maintained stable respiratory status thereafter. Venous blood gas was within normal limits with no hypoxemia.

Given her young age and rapid clinical improvement, whole-lung lavage was deferred. No repeat chest imaging was obtained before discharge because of her favorable clinical course. She was discharged on continued ERT and prophylactic antimicrobials (fluconazole and Bactrim) and immunoglobulin supplementation. At that time, her oxygen saturation remained normal on room air noted on multiple outpatient visits.

### 2.3. Follow-Up and Outcome

At 2 months of age, the patient underwent a successful 10/10 HLA-matched unrelated donor bone marrow transplant following a conditioning regimen of thymoglobulin and busulfan (AUC: 53.5 mg·h/L). ERT was discontinued prior to initiation of HSCT conditioning. At 4 months of age, the patient had normal oxygen saturation in room air and had a normal pulmonary exam and no ongoing symptoms. Repeat chest radiography showed no ground glass opacities. Donor chimerism analysis at 14 months post-transplant revealed stable mixed donor chimerism with 29% donor-derived CD33+ myeloid cells and 54% donor-derived CD3+ T cells. At the one-year post-HSCT immune evaluation, the patient also demonstrated evidence of immune reconstitution with normal numbers of total CD4+ T cells, naïve CD4+ T cells, B cells, and NK cells, as well as normal T cell proliferation to PHA, and freedom from immunoglobulin replacement. ADA enzymatic activity and concentration of toxic metabolites in red blood cells at one-year post-HSCT was improved compared to pre-transplant but did not reach the normal range.

## 3. Discussion

Pulmonary alveolar proteinosis (PAP) is a rare non-infectious pulmonary complication observed with increased frequency in ADA-SCID compared to other forms of SCID [3,4]. PAP is characterized by the accumulation of surfactant material within the alveoli due to impaired clearance by dysfunctional alveolar macrophages, a process linked to the underlying metabolic defect in ADA deficiency [5]. ADA deficiency leads to the accumulation of toxic purine metabolites, including deoxyadenosine and its phosphorylated derivatives (dAXP), particularly dATP, which inhibits ribonucleotide reductase, disrupts DNA synthesis, and contributes to cellular dysfunction. These metabolic disturbances, together with altered adenosine signaling, may impair alveolar macrophage function and surfactant clearance, promoting the accumulation of intra-alveolar surfactant material [1]. Clinically, PAP may manifest as progressive hypoxemia, respiratory distress, and radiographic evidence of diffuse alveolar opacification, and can contribute to significant morbidity if not promptly recognized and treated [6,7]. Treatment of PAP in the context of primary immunodeficiency is tailored to the underlying defect [8,9,10,11]. It includes enzyme replacement therapy (PEG-ADA) and definitive therapy like gene therapy or allogeneic hematopoietic stem cell transplantation (HSCT) for ADA–SCID [3,10,12]. Long-term pulmonary function outcomes in patients with ADA-SCID and PAP who have undergone bone marrow transplantation or other curative therapies are generally favorable, with most cases demonstrating histopathologic and clinical resolution of PAP and restoration of normal or near-normal lung function [3]. Although whole lung lavage (WLL) remains the standard of care for autoimmune PAP, its role in ADA-SCID is limited. WLL is a purely mechanical intervention that removes accumulated surfactant without correcting the underlying metabolic toxicity; in ADA-SCID, ongoing deoxyadenosine triphosphate accumulation would continuously impair alveolar macrophages and drive rapid surfactant re-accumulation, rendering lavage a temporizing measure at best. The procedure also carries substantial risk in immunocompromised neonates, requiring general anesthesia, endotracheal intubation, and ventilation in the setting of severe bilateral lung disease. Notably, no cases of WLL for PAP in ADA-SCID have been reported in the literature; by the time PAP was systematically recognized in this population, effective metabolic correction with ERT and HSCT had already demonstrated rapid and durable PAP resolution by addressing the root cause rather than the downstream consequence.

An important consideration in interpreting the patient’s rapid respiratory improvement is the temporal overlap between diagnostic BAL and escalation of ERT. Although a minor contribution from mechanical removal of alveolar material during BAL cannot be entirely excluded, the extensive and diffuse pulmonary involvement demonstrated on chest CT, together with the limited volume of fluid used during the procedure, makes a clinically significant therapeutic benefit from BAL highly unlikely. The patient’s rapid improvement following ERT dose escalation, along with sustained clinical and radiographic resolution, supports the potential role of metabolic correction in restoring alveolar macrophage function and surfactant homeostasis. Nevertheless, the relative contributions of these interventions cannot be definitively established from a single case.

In a single-center cohort, PAP resolved after HSCT, and long-term survivors generally had normal CD4 T cell numbers and function. However, the speed of PAP resolution also carries long-term implications. Prolonged PAP increases the risk of irreversible pulmonary fibrosis. Some patients developed chronic lung disease such as bronchiectasis or asthma, and late deaths from respiratory failure, indicating that pre-existing or persistent lung injury may influence outcomes [13]. Similarly, Grunebaum et al. reported 7 out of 16 ADA-SCID patients who developed PAP, and the pulmonary complication was reversed after either enzyme replacement therapy or allogeneic HSCT [3,14]. Only 2 (28.6%) of 7 patients with ADA deficiency with PAP are long-term survivors, which is similar (*p* = 0.14) to the 6 of 9 patients without PAP who survived. The time course of pulmonary recovery following treatment initiation was not described.

Although resolution of PAP following ERT and HSCT represents a favorable pulmonary outcome, long-term respiratory surveillance remains essential. Patients undergoing HSCT are at risk for late pulmonary complications, including bronchiolitis obliterans syndrome, recurrent pulmonary infections, and chronic structural lung abnormalities such as bronchiectasis or pulmonary fibrosis [15,16,17]. These complications may contribute to persistent or progressive respiratory impairment despite successful immune reconstitution. Therefore, longitudinal follow-up with pediatric pulmonology, including regular clinical assessments, age-appropriate pulmonary function testing when feasible, and chest imaging when clinically indicated, is important for early detection of late pulmonary complications [16].

The Primary Immune Deficiency Treatment Consortium (PIDTC) found that event-free and overall survival are highest when HSCT is performed early and in the absence of active infection but did not specifically report long-term pulmonary function in the PAP subset [18]. Definitive therapy for ADA-SCID has demonstrated durable immune reconstitution and decreased infection rates over long-term follow-up, with most patients remaining intervention-free and alive at 5–15 years post-treatment [19,20,21]. ADA-SCID should be conceptualized not merely as an immunodeficiency but rather as a multisystem disorder where untreated disease contributes to cumulative toxicity in multiple organ systems. The Polish national experience with ADA-SCID illustrates this principle: one patient with multiple organ failure died shortly after admission, before the diagnosis could be confirmed [17]. This case, along with the broader literature, reinforces that delayed diagnosis can result in irreversible multi-organ failure that no subsequent therapy can reverse. The rapid resolution of PAP observed in our case, from severe bilateral lung disease to complete radiographic clearing, exemplifies prompt recognition of ADA-SCID, immediate initiation of metabolic correction, and rapid resolution of what had been severe, life-threatening pulmonary disease, all occurring within a compressed timeframe of months rather than years. Overall, the management of ADA-SCID-associated PAP requires a multidisciplinary approach involving neonatology, pediatric pulmonology, and clinical immunology and oncology to ensure timely respiratory stabilization, diagnostic evaluation, and initiation of disease-specific therapy. Close coordination among these specialties, along with the transplant team, is essential to optimize the timing of interventions and facilitate the transition from acute respiratory management to definitive treatment with HSCT.

The clinical significance of early identification in ADA-SCID is highlighted by comparing our case with the recently published report by de Manuel Gómez et al. (2026), which described PAP in an ADA-SCID patient diagnosed through clinical presentation in a setting without universal SCID newborn screening [22]. The authors documented similar severe bilateral PAP with characteristic crazy-paving patterns on imaging with achieved radiographic improvement. However, the timeline to resolution diverged dramatically compared to our case: in the de Manuel Gómez et al. case, delayed diagnosis allowed prolonged accumulation of toxic purine metabolites before treatment, and PAP resolution was documented approximately 3 years after gene therapy initiation [22]. In the present case, newborn screening enabled rapid initiation of ERT within weeks of birth, and complete resolution of severe bilateral PAP occurred within months. This contrast likely reflects a dose–response relationship between the duration of untreated metabolic toxicity and the time required for pulmonary recovery.

## 4. Conclusions

This case demonstrates that PAP in ADA-SCID, even when presenting as severe bilateral lung disease, is potentially a fully reversible complication when metabolic correction is achieved promptly. The compressed clinical timeline, from life-threatening pulmonary compromise to complete resolution within months, powerfully illustrates the narrow but critical therapeutic window that exists before irreversible structural damage occurs. These findings reinforce the importance of universal newborn screening for SCID, immediate initiation of ERT upon diagnosis of ADA-SCID, and timely transition to definitive cellular therapy. Early identification and treatment can transform what would otherwise be a fatal or chronically debilitating disease into a treatable condition with improved long-term outcomes.

## Data Availability

The clinical data supporting the findings of this case report are contained within the article. Additional patient-level data is not publicly available to protect patient confidentiality.
